# Cavitation Feedback Control of Focused Ultrasound Blood-Brain Barrier Opening for Drug Delivery in Patients with Parkinson’s Disease

**DOI:** 10.3390/pharmaceutics14122607

**Published:** 2022-11-26

**Authors:** Yuexi Huang, Ying Meng, Christopher B. Pople, Allison Bethune, Ryan M. Jones, Agessandro Abrahao, Clement Hamani, Suneil K. Kalia, Lorraine V. Kalia, Nir Lipsman, Kullervo Hynynen

**Affiliations:** 1 Physical Sciences Platform, Sunnybrook Research Institute, Toronto, ON M4N 3M5, Canada; 2 Division of Neurosurgery, Sunnybrook Health Sciences Centre, Toronto, ON M4N 3M5, Canada; 3 Hurvitz Brain Sciences Research Program, Harquail Centre for Neuromodulation, Sunnybrook Research Institute, Toronto, ON M4N 3M5, Canada; 4 Department of Medicine, University of Toronto, Toronto, ON M5S 1A8, Canada; 5 Krembil Research Institute, University Health Network, Toronto, ON M5T 0S8, Canada; 6 Division of Neurosurgery, Toronto Western Hospital, Toronto, ON M5T 2S8, Canada; 7 Division of Neurology, Toronto Western Hospital, Toronto, ON M5T 2S8, Canada; 8 Department of Medical Biophysics, University of Toronto, Toronto, ON M4N 3M5, Canada; 9 Institute of Biomedical Engineering, University of Toronto, Toronto, ON M5S 3G9, Canada

**Keywords:** focused ultrasound, blood-brain barrier opening, cavitation dose, microbubble infusion, Parkinson’s disease

## Abstract

Magnetic resonance-guided focused ultrasound (MRgFUS), in conjunction with circulating microbubbles, is an emerging technology that can transiently enhance the permeability of the blood-brain barrier (BBB) locally and non-invasively to facilitate targeted drug delivery to the brain. In this clinical trial, the feasibility and safety of BBB modulation in the putamen were evaluated for biweekly therapeutic agent delivery in patients with Parkinson’s disease. The performance of the clinical MRgFUS system’s cavitation feedback controller for active power modulation throughout the exposures was examined. The putamen was targeted unilaterally by an ExAblate Neuro MRgFUS system operating at 220 kHz. Definity microbubbles were infused via a saline bag gravity drip at a rate of 4 µL/kg per 5 min. A cavitation emissions-based feedback controller was employed to modulate the acoustic power automatically according to prescribed target cavitation dose levels. BBB opening was measured by Gadolinium (Gd)-enhanced T1-weighted MR imaging, and the presence of potential micro-hemorrhages induced by the exposures was assessed via T2*-weighted MR imaging. A total of 12 treatment sessions were carried out across four patients, with target cavitation dose levels ranging from 0.20–0.40. BBB permeability in the targeted putamen was elevated successfully in all treatments, with a 14% ± 6% mean increase in Gd-enhanced T1-weighted MRI signal intensity relative to the untreated contralateral side. No indications of red blood cell extravasations were observed on MR imaging scans acquired one day following each treatment session. The cavitation emissions-based feedback controller was effective in modulating acoustic power levels to ensure BBB permeability enhancement while avoiding micro-hemorrhages, however, further technical advancements are warranted to improve its performance for use across a wide variety of brain diseases.

## 1. Introduction

Parkinson’s disease (PD) is a neurodegenerative disorder that mainly affects the motor system of more than 10 million people worldwide [1]. Patients with PD can experience tremor, rigidity and slow movement, as well as mental and behavioral changes, sleep problems, depression, memory difficulties, and fatigue. The motor symptoms are the result of reduced dopamine production in the brain’s basal ganglia. As dopamine replacement therapy, levodopa has become the standard of care for improving motor symptoms, but commonly with various long-term motor complications [2]. Another key hallmark of PD is the abnormal accumulation of a protein called alpha-synuclein in the brain, which leads to cellular toxicity and neurodegeneration [3]. Potential disease modifying treatments to reduce alpha-synuclein accumulation are limited by many drugs’ inability to cross the blood-brain barrier (BBB) [4]. A targeted, non-invasive delivery of potential drugs through the BBB may significantly improve the efficacy and safety profiles for treating PD.

Magnetic resonance-guided focused ultrasound (MRgFUS), combined with intravascular microbubbles (an existing ultrasound contrast agent for diagnostic imaging), is an emerging technology that can transiently enhance the permeability of the BBB locally and non-invasively [5]. Pre-clinical studies have demonstrated the safety and efficacy of microbubble-assisted MRgFUS for delivering various therapeutic agents across the BBB [6,7,8,9,10,11]. Several phase I clinical trials have been conducted since 2015, mainly to examine the procedural safety for various intracranial targets such as those associated with brain tumors [12,13,14,15], Alzheimer’s disease [16,17,18,19,20], PD [21,22], and amyotrophic lateral sclerosis (ALS) [23]. Here, we report on technical aspects of a phase I clinical trial of microbubble-assisted MRgFUS in patients with PD and mutations in GBA1 [24], in which a 55 kDa recombinant form of the human beta-glucocerebrosidase enzyme (encoded by GBA1) was delivered biweekly across BBB to the putamen, a key brain structure related to movement. Glucocerebrosidase addresses a key pathway deficient in PD patients with genetic predisposition and has been shown to be related to alpha-synucleinopathy [25]. Delivery of glucocerebrosidase or other enzymes to the affected brain structures is one potential strategy to reduce or prevent neurodegeneration in PD patients.

The mechanism of BBB opening via MRgFUS involves mechanical interactions between ultrasound and intravascular microbubbles flowing within capillary vessels in the focal volume, which induce stresses on endothelial cells and their tight junctions [26,27] that temporarily allow drug molecules to diffuse through vessel walls into the extravascular space, before barrier functions are restored up to 24 h later [28]. It has been demonstrated that microbubble-assisted MRgFUS BBB opening can be achieved safely with the use of appropriate treatment parameters, which include factors such as the ultrasound pressure level, frequency, pulse length, pulse repetition frequency, sonication duration, as well as the local microbubble concentration [29,30,31,32,33,34,35]. However, if excessive ultrasound parameters are employed (e.g., high pressure, long exposure duration), red blood cells (RBCs) can extravasate from capillary vessels and may induce hemorrhagic lesions within the focal volume [36]. For an eloquent target such as the putamen, it is particularly important to control the ultrasound exposures to avoid tissue damage.

The clinical MRgFUS brain system (ExAblate, InSightec) has incorporated a cavitation emissions-based feedback controller for active power modulation during ultrasound delivery. Here, pieozoelectric transducers detect pressure waves that microbubbles scatter and reradiate in response to ultrasound stimulation, or the so-called cavitation emissions. Spectral analysis of these cavitation emissions has been shown to be helpful in treatment monitoring and control in pre-clinical studies of microbubble-assisted MRgFUS [37,38,39,40,41]. This study investigates the performance of the cavitation feedback controller in a clinical setting, and discusses potential future improvements.

## 2. Materials and Methods

### 2.1. Study Design and Participants

This open label, prospective, proof-of-concept, phase I trial (clinicaltrials.gov identifier: NCT04370665) was designed to study the safety and feasibility of delivering beta-glucocerebrosidase (Cerezyme; Genzyme, Cambridge, MA, USA) to the putamen unilaterally in patients with PD. The treatment side was chosen based on the relative lateralized severity of the patient’s symptoms. The procedure was repeated once every two weeks for a total of 3 treatment sessions per patient. Bi-weekly scheduling was based on the recommended dosage on the drug label [42]. The study was approved by our institutional Research Ethics Board (project ID: 368–2018). All patients and their primary caregivers provided informed consent prior to study enrolment. Candidates were excluded from the trial if they were contraindicated to MRI or MR/ultrasound contrast agents, were at an increased risk of cerebral bleeding, or had active intracranial, cardiovascular, pulmonary, or renal disease. The inclusion and exclusion criteria, as well as primary outcomes, were reported previously [24]. This report focuses on technical details of FUS treatments.

### 2.2. MR-Guided Focused Ultrasound (MRgFUS)

The investigational MRgFUS device consisted of a hemispherical array of 1024 transducers operating at a central frequency of 220 kHz (ExAblate Neuro; InSightec Inc, Haifa, Israel). On the day of the procedure, a Cosman-Roberts-Wells (CRW) stereotactic frame was fixed to the patient’s head under local anaesthetic. The CRW frame was coupled to the helmet transducer array with the patient lying on the MR bed in supine, head-first position. A rubber membrane was fixed to the patient’s head and the helmet, allowing degassed water to circulate within the intervening space to provide acoustic coupling. A 3-Tesla MRI system (Magnetom Prisma, Siemens Healthineers, Erlangen, Germany) was used to obtain 2D T2-weighted (turbo spin-echo, TR: 6500 ms, TE: 98 ms, in-plane resolution: 0.9 mm × 0.9 mm, slice thickness: 2 mm) and 2D T2*-weighted (gradient echo, TR: 444 ms, TE: 20 ms, in-plane resolution: 0.9 mm × 0.9 mm, slice thickness: 3 mm) images with the body coil for treatment planning. Putamen structures were identified on axial T2-weighted images for targeting purposes. Pre-acquired CT images were co-registered with the intraoperative MR images for correcting skull-induced phase aberrations of the transmit beam [43]. In each treatment session, T2*-weighted images were acquired following a series of initial sonications with microbubbles to assess whether the target cavitation dose levels were appropriate for avoiding RBC extravasations. If suspected regions of signal hypointensity were detected in T2*-weighted MRI, cavitation dose levels were lowered for subsequent sonications. Following treatment, the stereotactic frame was removed and follow-up MR images were acquired with an 8-channel head coil for improved image quality. The MRI contrast agent Gadovist (1.0 mmol/mL; Bayer AG, Germany) was injected at a dose of 0.1 mL/kg, and 3D T1-weighted gradient-echo images (T1-MP RAGE, TR: 2000 ms, TE: 3 ms, TI: 900 ms, isotropic spatial resolution: 0.9 mm) were acquired following a delay of approximately 15 min to detect BBB opening. T2*-weighted images were re-acquired to monitor for indications of RBC extravasations produced within the target volumes. The MRI protocol was repeated one day following each treatment session to confirm restoration of BBB integrity.

Cerezyme was infused intravenously (dose at 15, 30 and 60 IU/kg for the 3 treatments, respectively) over one hour [42], which included the time for patient preparation, MR scanning, and target planning. Following baseline sonications, microbubbles (Definity; Lantheus, North Billerica, MA, USA) were infused intravenously via a saline bag gravity drip at an infusion rate of 4 μL/kg per 5 min. At the time of this trial, the maximum allowable dose of Definity microbubbles approved by Health Canada was 150 μL/kg. The number of vials of microbubbles, the saline bag volume, and the drip rate were adjusted to reach an in-vivo concentration of 4 μL/kg per 5 min according to Equation (1). The drip rate was measured continuously using an infusion rate monitor (DripAssist; Shift Labs, Seattle, WA, USA). A delay of five minutes between the beginning of microbubble infusion and the initial sonication allowed the microbubble concentration to reach a steady state in vivo. Subsequent sonications were carried out continuously with no delay.

(1) $$\text{Drip Rate}\;(mL/min)=\frac{concentration\;0.004\;(mL/kg)\times \text{body weight}\;(\text{kg})\;\times \;\text{saline volume}\;(\text{mL})}{\text{Definity}\;(\text{vials})\times 1.5(\text{mL}/\text{vials})\;\times \;5(\text{min})}$$

The ultrasound exposures for each sonication consisted of 10 ms pulses repeated once every second (1% duty cycle per target), for a total duration of 2 min. With these parameters, the MRgFUS system permitted exposure of an arbitrary-shaped grid in a single sonication with a maximum of 32 sub-spots (i.e., 32% effective duty cycle at maximum). The peak negative acoustic pressure at the focus was estimated to be on the order of 500 kPa at 5 W [11], although the estimated pressure was not used for exposure control. Acoustic power was controlled automatically by the cavitation-feedback controller. Eight cavitation receivers installed at different locations within the hemispherical helmet surface were used to detect cavitation signals during the sonications. Both narrow-band (110 ± 5 kHz) and broad-band (110 ± 40 kHz, 143 ± 30 kHz) spectral integrations around the subharmonic frequency were calculated in real-time. Individual sub-spots in a grid were sonicated sequentially during the 1 s pulse repetition period, and the mean of the cavitation signals across all sub-spots and all receivers was used to modulate the applied power level in a feedback control loop. With this feedback control approach, the spatial distribution of the resulting cavitation dose was often found to be heterogeneous amongst individual sub-spots, with a degree of heterogeneity proportional to the grid size. Considering both the relatively small coverage volume required and the importance of safety for the putamen target, multiple small grids of 2–5 sub-spots were employed in this study (sub-spot spacing: 2.5 mm) to cover the prescribed treatment volume. On average, 19 ± 5 grids (range: 11–25) were needed per treatment session to cover the unilateral putamen volume. The total sonication time in a treatment ranged from 22–50 min.

Cavitation activity was measured in the form of cavitation dose, which represents the product of the cavitation signal magnitude (units of volts) and the temporal duration (units of seconds). The cavitation magnitude at each time point (i.e., for each 10 ms pulse) was defined as the channel-mean Fourier spectrum subharmonic integration as described above. Cavitation dose was calculated by summing all time points of cavitation magnitudes and dividing by the number of sub-spots of that sonication. The feedback control loop employed in this study modulated the applied acoustic power in an attempt to deliver the target cavitation dose level at a constant rate in time across the total exposure duration. The power was ramped up until the corresponding mean rate was obtained across the entire grid (target cavitation dose level/2 min), after which the power level was modulated continuously for the remainder of the exposure. Baseline RF noise was present in the cavitation signals that was both acoustic power and pulse pattern dependent. Baseline acoustic signals were acquired at various power levels during MRgFUS system calibration, and were subtracted automatically from the cavitation signals measured during treatment on a power-wise basis using a lookup table. Despite this subtraction approach, a non-zero baseline cavitation level was sometimes detected with the patient setup (i.e., without circulating microbubbles). Patient-specific baseline cavitation dose levels were measured at fixed power levels of 5 W and 10 W before infusing microbubbles (10 ms pulses every 1 s, 2 min exposure duration), and were compensated for manually when prescribing target cavitation dose levels for sonications with microbubbles. For the putamen targets in this trial, baseline cavitation dose levels were either 0.00 or 0.01 across all treatment sessions, and therefore had a minimal impact on the prescribed target cavitation dose levels.

Target cavitation dose levels were chosen based on experience from earlier clinical trials at our institution in patients with brain tumors, Alzheimer’s disease and ALS [12,16,23]. In this study, the target cavitation dose level ranged from 0.20–0.40. Both a maximum acoustic power level and an instantaneous cavitation magnitude were pre-defined safety measures used to terminate sonications automatically if either were exceeded. The cavitation dose of each sub-spot was mapped spatially over the grid in real-time and displayed on the MRgFUS system console, under the assumption that cavitation signals received at a given time originated from the corresponding sub-spot being sonicated. If the cavitation dose was concentrated within less than half of the sub-spots in a grid, the sonication was terminated manually by the MRgFUS system operator once the target cavitation dose level was reached at those sub-spots. The grid was subsequently re-sonicated with the original hotspots removed to deliver the target cavitation dose level throughout the entire grid.

### 2.3. Outcome Measures

The primary outcomes were clinical and radiographic safety, as well as the technical feasibility of reversible and repeated BBB opening. Successful BBB opening and restoration was defined, respectively, as elevated signal intensity within gadolinium-enhanced T1-weighted MR images acquired immediately post-treatment and by the absence of such enhancement one day following treatment. Safety was measured by clinical exams during the procedure and at each follow-up visit, as well as radiographic examination for any adverse events including micro-hemorrhage or RBC extravasation (assessed via T2*-weighted MRI), swelling, or mass effect.

## 3. Results

**Figure 1:**
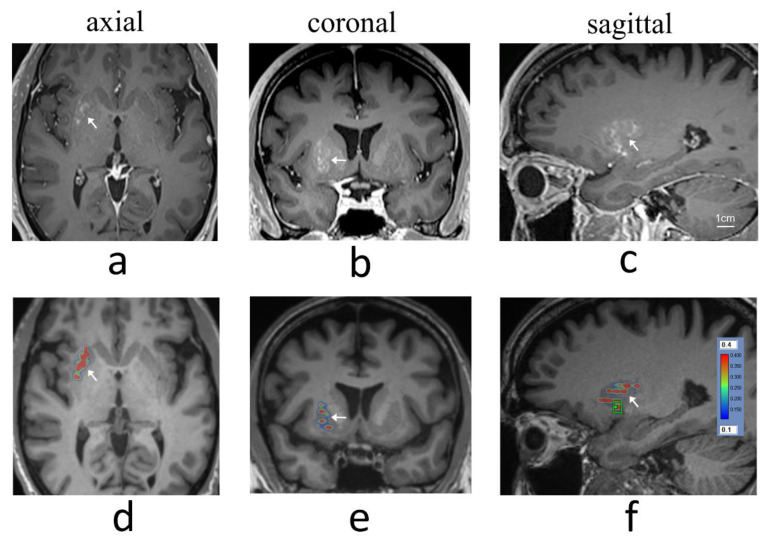
Gd-enhanced T1-weighted MRI showing BBB opening within the right putamen (arrows) immediately post-treatment in (**a**) axial, (**b**) coronal, and (**c**) sagittal views for treatment session 1 in patient 2 (cavitation dose target = 0.40). Images (**d**–**f**) show corresponding cavitation dose maps overlaid on pre-treatment T1-weighted planning MR images (green box in image (**f**) indicates the final sonication grid).

A total of 12 treatment sessions were carried out across four patients (age: 51 to 56) between September 2020 and February 2021, and were reported in Meng et al. [24]. BBB permeability within the targeted putamen was elevated successfully in all treatments, as revealed by Gd-enhanced T1-weighted MRI immediately post treatment (Figure 1). The Gd-enhanced signal intensity within the targeted volume was increased by 14% ± 6% relative to the untreated contralateral side (mean ± standard deviation). Measurement results from individual treatment sessions are provided in Table 1. No contrast enhancement was observed in the treated putamen on MR imaging scans acquired one day following each treatment session, indicating closure of the BBB.

**Figure 2:**
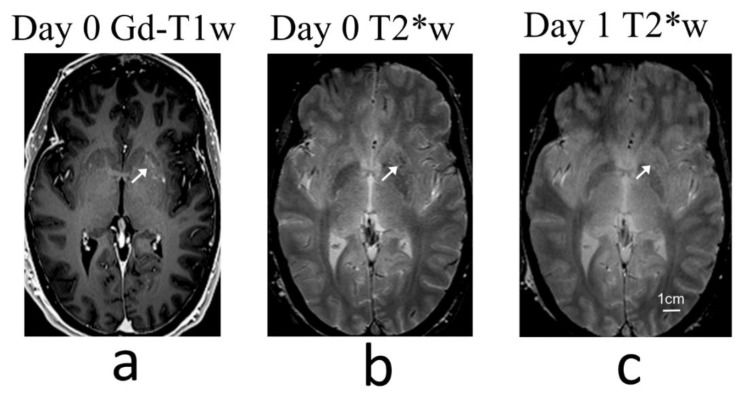
MRI summary of treatment session 1 in patient 1 (cavitation dose target = 0.40). (**a**) Gd-enhanced T1-weighted MR image shows BBB opening within the left putamen (arrows). (**b**) T2*-weighted MR images acquired immediately post-treatment revealed a small number of hypointense pixels within the target volume that were constrained within the axial slice shown. (**c**) Corresponding T2*-weighted MR image acquired one day post-treatment no longer contained regions of signal hypointensity within the targeted putamen.

An initial target cavitation dose level of 0.25 was applied in the first treatment session of the first patient. Intraoperative T2*-weighted MRI did not detect any abnormal signals, and the target cavitation dose level was increased to 0.40 for subsequent sonications in this treatment session. However, post-treatment T2*-weighted imaging with the head coil revealed a small number of isolated hypointense spots within the treated volume, which were resolved the next day on follow-up imaging (Figure 2). Although it was not definitively clear whether these hypointense T2*-weighted signals were caused by RBC extravasations, the target cavitation dose level was reduced to 0.35 and 0.30 in the two subsequent treatment sessions of this patient. For the second patient the target caviation dose levels were 0.40, 0.30 and 0.25, and in the final two patients a dose level of 0.20 was applied across all treatment sessions (Table 1).

**Figure 3:**
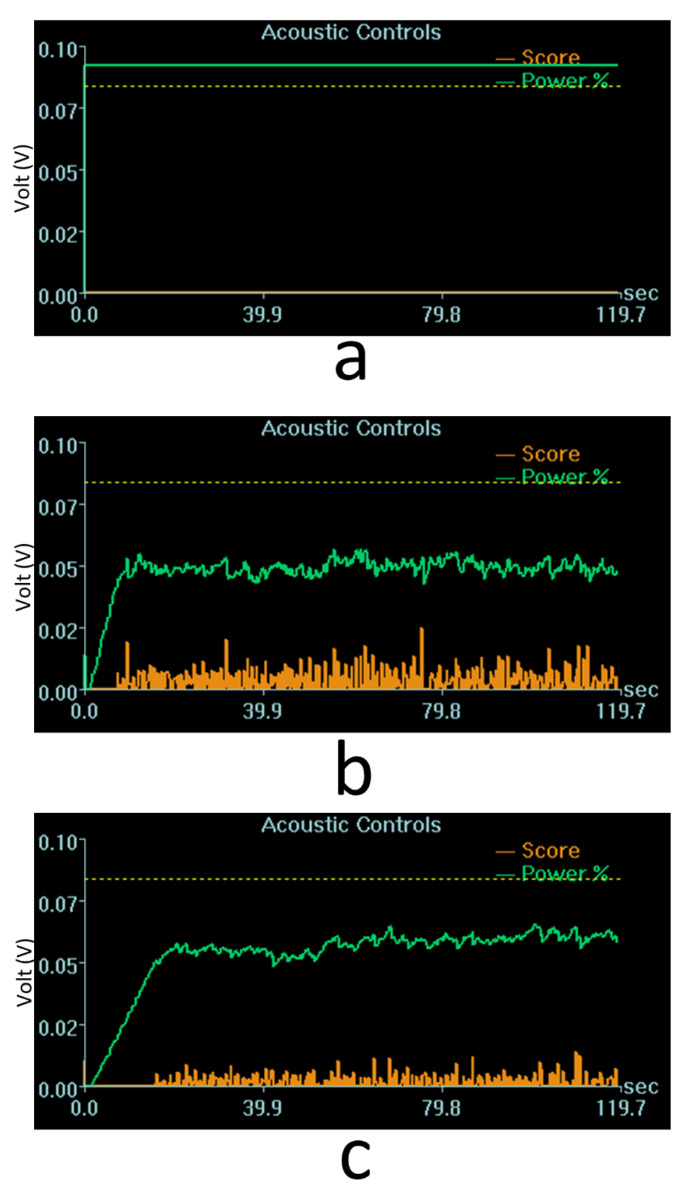
Representative plots of acoustic power (green lines) and cavitation (orange lines) levels as a function of time, as displayed on the MRgFUS system console. (**a**) Baseline sonication at 10 W (i.e., without circulating microbubbles; cavitation dose = 0.00). (**b**) Sonication with microbubbles in patient 2 (cavitation dose target = 0.40; maximum acoustic power = 6.1 W). (**c**) Sonication with microbubbles in patient 3 (cavitation dose target = 0.20; maximum acoustic power = 7.0 W).

On average, the maximum applied acoustic power was 6.1 ± 1.2 W (mean ± standard deviation) across a total 241 sonications, 48 of which (20%) were terminated manually by the MRgFUS system operator due to hotspot formation in the cavitation dose maps. The maximum applied power across individual treatment sessions is provided in Table 1. Two examples of cavitation signals with target dose levels of 0.40 and 0.20 are shown in Figure 3. It can be seen that the controller modulates the applied power automatically based on the target cavitation dose level. The applied power appeared to be affected more by the patient’s specific skull characteristics than by the prescribed target cavitation dose level. For instance, although the target cavitation dose level was higher in Figure 3b than in Figure 3c (0.40 vs. 0.20), the corresponding maximum acoustic power was lower in this case (6.1 W vs. 7.0 W) presumably due to differences in skull transmission between the two patients.

Lenticulostriate arteries are small perforating arteries measuring less than 1 mm in diameter that supply the putamen from its inferior aspects. They were clearly visible on Gd-enhanced T1-weighted MR images acquired immediately post-treatment, whereas corresponding arteries on the untreated contralateral side were only slightly visible due to partial volume effects with the imaging parameters employed (imaging resolution: 0.9 mm × 0.9 mm × 0.9 mm). In comparison, all cerebral arteries larger than approximately 1.5 mm in diameter were clearly visible in Gd-enhanced images. It is possible that the lenticulostriate arteries within the treated volumes were dilated slightly in size as a result of stresses exerted during the sonications, and therefore became more visible on Gd-enhanced T1-weighted MRI. In 5 of the 12 treatment sessions these arteries were still slightly visible on Gd-enhanced images acquired on day one, though a lack of signal enhancement in background tissue regions confirmed restoration of BBB integrity in these cases.

None of the patients experienced an adverse event over the course of this study. All patients were discharged on the same day after their procedures. This article focuses on technical descriptions related to cavitation emissions-based treatment guidance. Clinical results from this patient cohort were presented in a separate report [24].

## 4. Discussion

In earlier clinical trials at our institution, multi-point sonications consisting of 3 × 3 and 2 × 2 square grids were performed at fixed power levels following power testing obtained via separate ramp sonications [12,16,23]. With the improved capability of the MRgFUS system to enable sonication of larger grids of up to 32 sub-spots, covering large treatment volumes within a practical timeframe has become feasible. However, running a ramp sonication at a single sub-spot for power testing over a large grid can lead to non-uniform cavitation responses due to tissue heterogeneity. Before the ability to perform ramp sonications at each sub-spot and to modulate the power level of each sub-spot individually was implemented on the clinical MRgFUS system, a different power modulation approach was developed that controls the mean cavitation dose level across the entire grid throughout the exposure duration. This cavitation emissions-based feedback controller has been adopted in all our ongoing clinical trials beginning in late 2019, including studies on glioblastoma (GBM), Her2-positive brain metastases, Alzheimer’s disease, as well as this trial on PD. Since the current trial was the only one in which the target was in a relatively consistent centralized brain location, and relatively small grids were employed in practice, it provided an opportunity to examine the performance of the controller in an ideal clinical setting.

In this study the ultrasound pulsing scheme for each sub-spot was fixed (i.e., 10 ms pulses at a pulse repetition frequency of 1 Hz for a total of 2 min), therefore the cavitation controller effectively regulated the mean cavitation magnitude over the sonication duration. Based on our experience from previous clinical trials, BBB opening can be achieved at low cavitation magnitudes in targets associated with Alzheimer’s disease, though higher cavitation levels are required in GBM patients, which increases the risk of inducing RBC extravasations. For the putamen targets in this trial, we operated conservatively, favouring safety over the level of enhanced BBB permeability, and therefore prescribed relatively low target cavitation dose levels (range: 0.20–0.40). Analyzing the data from all 12 treatment sessions, there was low correlation between the cavitation dose and Gd enhancement levels (R^2^ = 0.02, Table 1), potentially because the range of cavitation dose levels investigated was too narrow, the delay time between treatment and Gd-enhanced MR imaging was variable, or due to measurement noise from a small sample size. If higher cavitation levels are assumed to indicate stronger ultrasound-microbubble interactions, they should translate to a higher degree of BBB permeability and an increased risk of RBC extravasations. However, if a vasoconstriction effect is induced [44] that subsequently reduces blood perfusion within the focal volume, the resulting drug deposition over several hours post-treatment may not necessarily be higher than that obtained with lower cavitation levels that do not produce vasoconstriction. Furthermore, measurements of Gd enhancement immediately post-treatment do not necessarily correlate with the total drug deposition level, due to variations in the timing of Gd injection along with the relatively short half-life of Gd in circulation. For drugs with longer plasma half-lives, biomarkers with similar intravascular half-lives, or labeled radiotracers in combination with SPECT imaging can provide a more accurate measure of drug delivery efficacy [45].

**Figure 4:**
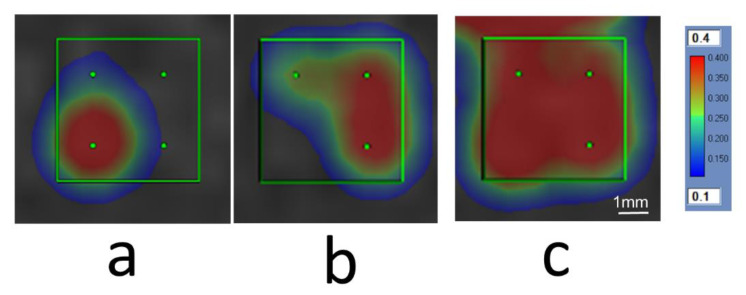
An example showing interactive operation to improve homogeneity of the cavitation map. (**a**) The sonication over a 2 × 2 grid generated one hotspot that reached the targeted cavitation dose of 0.4 while the other 3 subspots gained minimum dose. The sonication was terminated by the operator at 60 s. (**b**) The grid was re-sonicated by removing the hotspot that already reached the dose target level. (**c**) The accumulated dose map displays the sum of the two sonications. All four subspots reached the targeted cavitation dose more homogeneously. Note the cavitation dose above the grid in (**c**) was from a nearby treatment grid.

Despite the use of relatively small grids in this study (2–5 sub-spots), spatially heterogeneous cavitation dose distributions were observed frequently because the controller modulated the applied power level based on the average cavitation dose level across the entire grid. Approximately 20% of all sonications were terminated manually by the MRgFUS system operator because one or two sub-spots had reached the prescribed dose level while other sub-spots had not accumulated any dose (Figure 4). Although the remaining 83% of sonications were not terminated due to hotspot formation, many of these grids were associated with spatially heterogeneous cavitation dose distributions upon exposure completion. For example, a grid of 4 sub-spots with a prescribed target cavitation dose level of 0.40 was likely to result in two sub-spots obtaining a dose of 0.50, and the other two obtaining a dose of 0.30. This is one potential factor contributing to the variability of Gd enhancement measured within the target volumes across different treatment sessions (Table 1), in addition to the sub-spot spacing employed and the enhancement of lenticulostriate arteries. A cavitation imaging-based feedback controller with sub-spot control has been shown to perform well in animal studies with a clinical-prototype transmit/receive hemispherical phased array system [46]. In the future, it is anticipated that the implementation of individual sub-spot control within the clinical MRgFUS brain system will substantially improve treatment efficiency and reduce the risk of unwanted damage, particularly in cases where the target tissue volumes are heterogeneous.

The putamen is relatively central within the brain, which is an ideal location for MRgFUS treatment with a hemispherical transducer array. This is particularly relevant in the context of cavitation monitoring with the current clinical system, which uses a relatively small number of receivers (i.e., *n* = 8) distributed over the hemispherical surface for cavitation detection. In MRgFUS treatments of GBM and Her2-positive brain metastases, tumor locations are often found in peripheral brain regions close to the skull bone. Due to the limited electronic steering capabilities of the current 220 kHz clinical MRgFUS system (2.5 cm maximum distance from the array’s geometric focus), the array needs to be positioned off-center relative to the patient’s head such that the geometric focus is close to targets. As a result, the incident angles of the cavitation receivers relative to the skull curvature can vary significantly across different detectors. Using a spectral average across different receivers may introduce large errors in the measured cavitation magnitude in such scenarios. Passive cavitation mapping [47] with large-aperture 2D receiver arrays has been shown to enable 3D reconstruction of cavitation signals with high spatial resolution and improved localization accuracy [46,48,49,50,51]. A similar technique has been implemented on the 650 kHz clinical MRgFUS system for echo-focusing during thermal ablation treatments [52]. If passive cavitation mapping could be incorporated within the low-frequency device, the performance of the system’s cavitation feedback controller could be greatly improved. The receivers integrated within the current ExAblate system are tuned to the first subharmonic of the driving frequency (i.e., f/2 = 110 kHz). As such, only subharmonic signals and broadband spectral content near this frequency are currently used for cavitation feedback purposes with this system. The main advantages of using subharmonic signals for cavitation feedback control are that they are specific to nonlinear microbubble emissions and their low frequency reduces the impact of skull-induced aberrations [49]. Alternatively, the use of both ultraharmonic [39] and harmonic [40] signal content has been investigated for feedback control during BBB opening procedures. One advantage of using harmonic signals is that these microbubble emissions are typically induced at lower power levels than those required to induce other nonlinear cavitation signals (e.g., subharmonics, ultraharmonics). However, since harmonic signal content can also arise from other sources (e.g., nonlinear propagation in tissue), baseline sonications (i.e., without microbubbles in circulation) are required to separate background signals from microbubble activity [40]. It remains to be seen which feedback control approach works best in a practical clinical setting.

Microbubble infusion via saline bag gravity drip was employed in this study to produce a more consistent concentration in vivo than that obtained using bolus injections. Sonications were delivered consecutively without the need to time the start of exposures with the bolus arrival as in previous trials. An infusion rate of 4 μL/kg per 5 min was chosen to be consistent with previous trials using bolus injections [12,16,23]. An increased total microbubble dose of 150 μL/kg was approved by Health Canada prior to the beginning of this trial, which permits a maximum of approximately 3 h of continuous infusion and treatment time. In practice, the number of vials of microbubbles, the saline bag volume, and the drip rate were optimized based on the estimated treatment time for a particular patient. For example, to cover the putamen unilaterally in this study, an average of one hour of treatment time (~30 sonications) was anticipated. Therefore, a 150 mL saline bag was prepared (i.e., by removing 100 mL from a 250 mL saline bag) with a drip rate of approximately 2 mL/min in mind. For a patient with a body weight of 80 kg, 3 vials of microbubbles were mixed within the 150 mL saline, resulting in a drip rate of 2.13 mL/min based on Equation (1). Using standard 15 drops/mL tubing, the drip rate in this case corresponds to 32 drops/min. The drip rate was controlled precisely with the help of a drip rate counter. Drip rates were checked every 5–10 min, and adjusted if found to have deviated from the intended value. Microbubble infusion was paused during intraoperative MR imaging to minimize the total administered dose. A delay of two minutes following infusion re-initiation was enforced prior to beginning the sonication following each pause for MR imaging. This delay was shorter than the initial 5 min delay enforced prior to the first sonication in each patient since the long IV extension tubing was already primed at this point, and therefore microbubbles entered vascular circulation immediately upon re-initiation of infusion. In this study, the maximum number of sonications in a single treatment session was 36, which translated into approximately 80 min of infusion time (including delays after initiations of infusion), and a total microbubble dose of 64 μL/kg, less than half of the maximum dose limit. Infusion rates lower than 4 μL/kg per 5 min could be applied to reduce the total microbubble dose if needed, however, previous pre-clinical work with this MRgFUS system showed more sporadic cavitation signals and less consistent BBB opening at a dose of 2 μL/kg using bolus injections [11]. It remains to be seen whether microbubble infusion can produce better results than bolus injections at a lower concentration with an improved cavitation feedback controller.

Our study has several limitations. First, the sample size is relatively small with only 12 treatment sessions across 4 patients. A larger sized trial is necessary to further assess the safety and efficacy of drug delivery for this target. Second, the microbubble concentration and ultrasound exposure parameters were fixed in this study, and thus only one specific set was tested. Nevertheless, it is anticipated that these optimized parameters will become the standard set employed on this MRgFUS system in the foreseeable future. Third, the clinical MRgFUS system is under rapid development, including upgrades to both hardware and software. It is likely that cavitation signals measured by future systems are considerably different from those discussed in this paper. Because cavitation signal is not an international standard measure, such as temperature used for guiding thermal ablation treatments, it relies on system-specific calibrations for a particular setup. Therefore, current cavitation dose values may not be directly comparable to measurements on future MRgFUS systems. Attention should be paid to relative characteristics of cavitation signals rather than absolute values. Lastly, in this phase I trial the drug infusion began prior to the first sonication in each patient to separate potential drug-related adverse events from those related to FUS exposures. However, the half-life of the drug’s plasma enzymatic activity has been shown to range between 3.6 and 10.4 min [42]. Therefore, the in-situ drug concentration at the time of sonication was much lower than the temporal-peak value. To obtain the best therapeutic results when infusing drugs with short half-lives, it is most likely optimal to start the infusion concurrently with the initial sonication in each patient, a scheme that will be investigated in future clinical trials once the safety profile of the treatment procedure has been well established.

In conclusion, this study demonstrated the successful application of microbubble-assisted MRgFUS for BBB opening in the putamen to facilitate biweekly therapeutic drug delivery in patients with PD. Repeated unilateral BBB modulation of the putamen was performed safely, reversibly, and with good targeting accuracy and spatial coverage. The cavitation emissions-based feedback controller was effective in modulating acoustic power levels to ensure reliable BBB permeability enhancement while avoiding micro-hemorrhages, however, further technical advancements are warranted to improve its performance for use across a wide variety of brain diseases.

## Figures and Tables

**Table 1:** Technical parameters and measurements across all treatments. Acoustic power uncertainties denote one standard deviation from the mean. Gd enhancement was quantified relative to the untreated contralateral side.

	Dose Target Level	Total Sonication Numbers	Number of Sonications Terminated by Operator	Maximum Acoustic Power (W)	Gd Enhancement
Patient 1, Treatment 1	0.40	11	0	4.8 ± 0.6	11% ± 4%
Patient 1, Treatment 2	0.35	17	3	4.5 ± 0.6	6% ± 4%
Patient 1, Treatment 3	0.30	23	15	4.5 ± 0.4	19% ± 6%
Patient 2, Treatment 1	0.40	25	6	6.3 ± 0.6	23% ± 3%
Patient 2, Treatment 2	0.30	18	1	5.5 ± 0.4	14% ± 7%
Patient 2, Treatment 3	0.25	22	3	5.7 ± 0.6	27% ± 10%
Patient 3, Treatment 1	0.20	19	4	6.0 ± 0.6	12% ± 7%
Patient 3, Treatment 2	0.20	24	8	6.7 ± 0.6	12% ± 5%
Patient 3, Treatment 3	0.20	26	4	6.6 ± 0.6	15% ± 7%
Patient 4, Treatment 1	0.20	13	0	6.9 ± 1.1	13% ± 7%
Patient 4, Treatment 2	0.20	20	0	7.0 ± 1.0	7% ± 4%
Patient 4, Treatment 3	0.20	23	4	7.6 ± 1.4	12% ± 6%
